# Saved by the SPY*: Ulnar Artery Reconstruction With LCFA Graft for Hypothenar Hammer Syndrome

**Published:** 2020-08-05

**Authors:** Zachary S. Gala, Haripriya Ayyala, Stephen L. Viviano, Ashley Ignatiuk

**Affiliations:** Department of Surgery, Division of Plastic and Reconstructive Surgery, Rutgers New Jersey Medical School, Newark

**Keywords:** hypothenar hammer syndrome, lateral femoral circumflex artery (LCFA) graft, ulnar artery thrombosis, intraoperative SPY, intraoperative ICG

## Abstract

**Introduction:** Hypothenar Hammer syndrome refers to thrombosis/aneurysm of ulnar artery at Guyon's canal in wrist, with resultant arterial insufficiency in the ulnar artery distribution.^1^ Patients typically describe unilateral symptoms in the fourth and/or fifth fingers of the hand. Symptoms can range from asymptomatic to pain, pallor, paresthesia, weakness, cold intolerance, and eventually ulceration, necrosis, and gangrene of the distal digits.^1^ Treatment options range from conservative, lifestyle management, to medication, and ultimately to surgical intervention.

In this case report, we outline the second successful lateral circumflex femoral artery (LCFA) graft reconstruction of the ulnar artery in the setting of Hypothenar Hammer Syndrome conducted by the senior author. However, during this procedure, the use of intraoperative intravenous (IV) injection of indocyanine green (ICG) dye (hereafter ICG) imaging helped identify an additional area of stenosis previously unseen on pre-operative MRA, therefore enabling us to perform a more adequate resection and repair. To our knowledge, the use of intraoperative ICG for Hypothenar Hammer Syndrome and/or ulnar artery reconstruction has not been documented in the literature.

## CASE DESCRIPTION

A 62-year-old right-hand-dominant man presented with “few years” history of progressively worsening symptoms along the right fourth and fifth fingers, which included pallor, discoloration, pain, paresthesia, “pins and needles,” skin lesions, and ulcerations (that had since healed at surgical evaluation appointment). Magnetic resonance angiography (MRA) showed a 2- to 3-cm segmental occlusion at Guyon's canal and an incomplete superficial arch. After failed conservative management, operative intervention was planned. The authors performed a right-sided ulnar artery reconstruction with a lateral femoral circumflex artery (LCFA) arterial graft and sympathetctomy. Intraoperative indocyanine green (ICG) imaging revealed a larger area of stenosis previously unseen on the preoperative MRA scan. This resulted in the need for a larger incision, diseased artery segmental excision, and ultimately a larger LCFA graft. Since the pathologic segment was larger than previously thought, this enabled a more adequate surgical intervention that otherwise would have been insufficient based on MRA alone. Microsurgical anastomosis was performed, and ICG imaging revealed patent vessels.

See Supplemental Digital Content Figures (available at:

**Supplementary Figure S1 (Figure 1):** Diseased segment of the right (R) ulnar artery

**Supplementary Figure S2 (Figure 2):** Intraoperative SPY showing diseased segment of ulnar artery

**Supplementary Figure S3 (Figure 3):** LCFA isolation and graft preparation

**Supplementary Figure S4 (Figure 4):** Repaired segment of right (R) ulnar artery with LCFA graft in-place).

## QUESTIONS

1. What is hypothenar hammer syndrome?
2. How is hypothenar hammer syndrome typically treated?
3. Why the intraoperative ICG use? What is the big deal?
4. Should surgeons consider using intraoperative ICG more routinely?

## DISCUSSION

Hypothenar hammer syndrome refers to thrombosis/aneurysm of ulnar artery at Guyon's canal in wrist, with resultant arterial insufficiency in the ulnar artery distribution. This condition was first described by Van Rosen in 1934. Overall, the disease is quite rare, with about a 1.6% incidence rate, and a male predominance of M:F = 9:1. The term “hypothenar hammer” syndrome was coined by Conn et al in 1970,^2^^-^6 in which the hook of the hamate bone acts as an anvil for the ulnar artery, which is subject to repetitive forces (the hammer). The cause, for all intents and purposes, is effectively trauma. Risk factors include repeated vibration and occupations such as carpenters and mechanics. Ferris and Stone's^7^ landmark study suggested that underlying vascular anomalies, such as intimal hyperplasia, can predispose individuals to developing this disease. Patients typically describe unilateral symptoms in the fourth and/or fifth fingers of the hand. Symptoms can range from asymptomatic to pain, pallor, paresthesia, weakness, cold intolerance, and eventually ulceration, necrosis, and gangrene of the distal digits. If an aneurysm is present, some patients may present with a pulsatile mass. Workup usually contains a positive Tinel's test, in which tapping over the ulnar artery distribution elicits pain. However, some studies have shown that up to 17% of patients with this condition have normal Allen's tests.^8^ Workup is confirmed with contrast imaging (magnetic resonance angiography [MRA]) that shows segmental occlusion or aneurysm in the ulnar artery and a resultant incomplete superficial arch.

Treatment options range from conservative lifestyle management to medication and, ultimately, to surgical intervention. Regimens typically begin with nonoperative lifestyle management: smoking cessation, avoidance of recurrent trauma and exacerbating factors, or the use of padded/protective gloves. Medical treatments are the second step and traditionally target the Raynaud's-like phenomenon with the use of calcium channel blockers (CCBs) and antiplatelet (anti-PLT) medications.^1^^,^^5^^,^^6^ If these interventions are unsuccessful, medical professionals proceed to surgical treatment. Endovascular fibrinolysis is indicated for thrombotic lesions without aneurysm that have been present for less than 2 weeks. The most common operative treatment is arterial ligation and reconstruction. This procedure is indicated for patients with a digital-brachial index less than 0.7 and if conservative treatment measures have failed.^1^ The last-resort effort is typically the Leriche procedure, which is resection of the diseased arterial segment without reconstruction, indicated if the digital-brachial index is greater than 0.7. Surgical treatments typically consist of dissection and resection of the diseased arterial segment with arterial reconstruction. The repair was first done by end-to-end anastomosis of the ulnar artery. Lifchez and Higgins's 2009 study showed that venous graft reconstruction showed better long-term outcomes compared with end-to end anastomosis of the ulnar artery.^9^ Dethmers^10^ and Endress^11^ showed that most venous grafts used in ulnar artery reconstruction were occluded at various long-term study endpoints. Temming first described the use of a lateral circumflex femoral artery (LCFA) arterial graft for ulnar artery reconstruction of the ulnar artery in the setting of hypothenar hammer syndrome^12^^,^^13^. Ultimately, in 2017, De Niet showed that arterial grafts had better outcomes when compared with venous grafts in terms of long-term patency.^14^ At a 63-month follow-up, 11 of 11 grafts were patent, and 9 of 11 patients showed clinical improvement for LCFA reconstruction of the ulnar artery.

To our knowledge, the use of intraoperative ICG imaging has not been previously described. There are no true indications for the use of intraoperative ICG, but its postoperative use and efficacy have been well described. The authors decided to use ICG imaging before arterial ligation and sympathetctomy. This revealed an additional area of stenosis in the ulnar artery previously unseen on the preoperative MRA scan. Because the pathologic segment was larger than originally thought, a more radical dissection, ligation, sympathectomy, and LCFA graft ensued. Although the procedure was more drastic, it was also more appropriate, as it successfully identified all pathologic segments of the artery. If the authors solely used MRA, there would have been an incomplete resection of the ulnar artery, and it is likely that the patient's symptoms would not have completely resolved.

The authors would strongly consider using intraoperative ICG imaging in future cases to ensure that all pathologic segments were correctly identified and that an adequate surgical intervention was planned. More research needs to be done, however, as to the success and efficacy of intraoperative ICG compared with preoperative MRA alone.

## SUMMARY

A 62-year-old male carpenter with a long history of pain, pallor, discoloration, and skin lesions along the right fourth and fifth fingers was diagnosed with hypothenar hammer syndrome. MRA revealed a 2- to 3-cm segmental occlusion in the distal ulnar artery by Guyon's canal and an incomplete superficial arch. After failed treatment, the authors planned for resection of the pathologic segment, repair with an LCFA graft, and sympathetctomy. Intraoperative ICG imaging revealed an additional area of stenosed artery, previously unseen on the MRA scan. This newfound pathologic segment of artery called for a more extensive dissection, sympathectomy, arterial ligation and excision, and ultimately a larger LCFA graft. Although the resultant procedure was more drastic than previously planned, it was also more adequate for this patient's pathology. Without the intraoperative ICG imaging, no additional area of stenosis would have been seen, and the surgical management would likely have only been partially adequate. Microsurgical anastomosis was performed and postoperative ICG imaging revealed patent vessels.
